# Analytical Modeling of Wave Absorption Performance in Gradient Graphene/Polymer Nanocomposites

**DOI:** 10.3390/ma17122946

**Published:** 2024-06-16

**Authors:** Qin Zhao, Fang Li, Jili Liu

**Affiliations:** Hubei Key Laboratory of Theory and Application of Advanced Materials Mechanics, School of Science, Wuhan University of Technology, Wuhan 430070, China; 18271843289@163.com

**Keywords:** graphene/polymer nanocomposites, gradient distribution, wave absorption performance, equivalent performance

## Abstract

Due to the low impedance matching caused by the high dielectric permittivity of graphene, the strong absorption of electromagnetic waves by graphene/polymer nanocomposites is challenging. In this paper, an analytical model for microwave absorption based on Maxwell’s equation and the effective medium theory, considering the interface effect, was constructed to explore the effect of the gradient distribution of graphene in the polymer matrix on its microwave absorption performance. The outcome indicated that the impedance of the composites matched well with the air, and its attenuation ability for electromagnetic waves was obviously improved as the graphene concentration was distributed in a gradient form. For instance, when the thickness of the material is 10 mm, based on the optimal concentration of the homogeneous composites being 0.7 wt%, the graphene concentration range of the gradient composites is set to 0.7–0.9 wt% and distributed in three gradient forms of linear, parabolic, and 0.5 power. The results show that the microwave absorption performance is significantly improved compared with the homogeneous composites. Among them, the effective bandwidth on the 0.5 power distribution is 5.2 GHz, 0.5 GHz higher than that of the homogeneous composites. The minimum reflection loss (RL) is as low as −54.7 dB, which is 26.26 dB lower than that of the homogeneous composites. This paper contributes to the design and application of gradient absorbing structures.

## 1. Introduction

Wireless communication networks and electronic devices, which rely on the transmission and reception of electromagnetic waves, have been extensively used in both commercial and personal settings, significantly enhancing personal life quality and societal productivity. However, the increasing use of such electromagnetic wave-based devices has led to severe electromagnetic pollution, adversely affecting the efficient operation of electronic systems. Sensitive components in military equipment, such as aircraft and radar, can be disrupted or even damaged by electromagnetic pollution. Additionally, electromagnetic pollution has been linked to various health issues, including cancer and cardiovascular diseases [1,2,3]. Wave-absorbing materials have garnered significant attention from both academia and industry as a solution to mitigate electromagnetic pollution. Nevertheless, this solution requires materials with properties such as light weight, low density, strong absorption, and a broad effective absorption bandwidth. Further development is needed to address the limitations of existing wave-absorbing materials [4,5,6,7].

Graphene, a typical carbon nanomaterial, has attracted extensive attention in the field of microwave absorption due to its numerous advantages in absorbing electromagnetic waves [8]. Compared to magnetic materials, graphene exhibits superior dielectric properties, lower density, and better corrosion resistance [9]. Additionally, it possesses a larger specific surface area and better electrical conductivity than other carbon materials [10]. Furthermore, oxidized graphene contains numerous dangling bonds and defects within its crystal structure, significantly enhancing the interfacial polarization and dipole polarization effects of the composite material, facilitating electron transfer, and thereby improving the conductivity of the composite material. Consequently, graphene is considered an excellent microwave absorber [8,11]. However, achieving a high dielectric constant while maintaining lightweight properties in a single composite is challenging [12]. Microwave-absorbing materials with a polymer matrix offer advantages such as softness, light weight, excellent mechanical properties, and strong corrosion resistance. Moreover, composites formed by combining graphene with a polymer matrix can prevent graphene agglomeration and ensure effective dispersion. The presence of interface defects also positively affects the interface between graphene and the polymer, aiding in the formation of a conductive network, leading to a dual electromagnetic wave loss mechanism of conductivity loss and interface polarization loss [13,14]. Therefore, adding a small amount of graphene filler to the polymer matrix can achieve good dielectric properties while maintaining the lightweight nature of the absorbing material. Additionally, the electromagnetic parameters of composite materials can be controlled by adjusting the concentration of graphene, thus affecting the electromagnetic wave absorption performance of the composites. For instance, De Bellis G et al. [15] studied the effect of varying graphene filler concentrations on the electromagnetic properties of graphene/epoxy vinyl ester composites and found that the dielectric permittivity and conductivity of the composites increased with higher graphene concentrations. Ren et al. [16] fabricated wave-absorption composites using epoxy isocyanate as the matrix and graphene nanosheets (GNSs) as the wave-absorption filler. They examined the wave-absorption performance of these composites in the frequency range of 2.6–12.4 GHz. Composites with a GNS concentration of 3 wt% and a thickness of 3 mm exhibited favorable wave-absorption performance, with an effective bandwidth frequency of 5.8–6.6 GHz and a peak reflection loss of −15.7 dB. Increasing the thickness shifted the peak reflection loss from 10.2 GHz to 4.5 GHz, with a minimum reflection loss of −21.4 dB at 4.5 GHz. Li et al. [17] constructed graphene with a 3D network using a bubble template method and utilized it to fabricate graphene/polydimethylsiloxane rubber composites with excellent electromagnetic shielding performance. When the graphene concentration was 18.1 wt% and the composite thickness was 2 mm, the electromagnetic shielding performance reached −86 dB. Evidently, the wave-absorption performance of composites is significantly affected by the graphene concentration and thickness.

Graphene/polymer nanocomposites exhibit commendable wave-absorption properties. However, due to the highly dielectric nature of graphene, impedance matching is often unsatisfactory, hindering the incidence of electromagnetic waves [18]. Consequently, many researchers have developed composites with graphene as the absorbing agent in multi-layer structures to improve impedance matching and thereby enhance absorption capabilities. Min et al. [19] fabricated GNSs/epoxy resin composites with double- and single-layer absorbers, discovering that the double-layer absorbers exhibited more satisfactory reflection loss values and absorption bands compared to single-layer absorbers in the 2–18 GHz frequency range. Lu et al. [20] used multilayer graphene and polyethylene terephthalate (PET) to fabricate graphene/PET composites, achieving a maximum wave absorbance of 95.82% at 25.7 GHz and an average shielding efficiency of 19.14 dB in the 18–26.5 GHz range. Furthermore, the shielding efficiency of the composites gradually increased with the transition from a single- to a four-layer construction. Graded distributed graphene is advantageous for enhancing the wave-absorption performance of composites. For example, as reported by Zhang et al. [21], reduced graphene oxide (RGO)/polyurethane composites with a gradient distribution of RGO exhibited satisfactory wave-absorption performance. When the electromagnetic wave was incident from the surface with low graphene concentration, the reflection loss of the composites was −30 dB across the entire X-band, and over 99.5% of the wave was absorbed.

The impedance matching challenge can be addressed by constructing graphene/polymer nanocomposites with a gradient distribution of graphene. Unfortunately, literature on graphene/polymer nanocomposites with a gradient distribution of graphene is scarce, and the theoretical research is extremely rare. To our knowledge, for the research on the absorbing properties of absorbing materials at present, some scholars [22,23] have measured the dielectric constant of the material through experiments and then calculated the reflection loss by using the impedance of the material and free space. Other scholars have directly measured the reflection loss of the material through experiments [24,25]. Therefore, the mechanism by which gradient-distributed graphene affects the wave-absorption performance of graphene/polymer nanocomposites remains unclear and needs further elucidation.

In this study, based on the effective medium theory, we introduced the gradient distribution function of graphene concentration, considered the influence of the interface effect between graphene and polymer, and derived the modified gradient equivalent dielectric theory. Combining this with Maxwell’s theory in electromagnetic solid mechanics, we established a theoretical absorption model for graphene/polymer nanocomposites with a gradient distribution of graphene concentration. The influence of graphene concentration on the absorption properties of uniform composites was analyzed, and the optimal graphene concentration for composites with different thicknesses was determined. Furthermore, the impact of the gradient distribution mode of graphene concentration on the impedance matching and attenuation constant was studied to explore its effect on the absorption properties of gradient composites.

## 2. Analytical Model for Electromagnetic Wave Absorption

According to the electromagnetic wave absorption mechanism, wave-absorbing materials are based on fundamental principles: (1) achieving impedance matching between the material and air, which helps reduce the reflection of electromagnetic waves at the interface; (2) ensuring that the material’s ability to attenuate electromagnetic waves is sufficient to achieve full absorption.

The impedance matching ratio (*D*) of absorbing material to air is related to the relative complex permeability ($\mu_{r}$) and dielectric permittivity ($\varepsilon_{r}$) of the material, which can be calculated according to the following equations [26]:

(1) $$D=Z_{1}/Z_{0},$$

(2) $$Z_{1}={\left(\mu_{r}/\varepsilon_{r}\right)}^{1/2}Z_{0}.$$

where *Z*_1_ and *Z*_0_ are the impedance of material and the free space, respectively.

**Figure 1:**
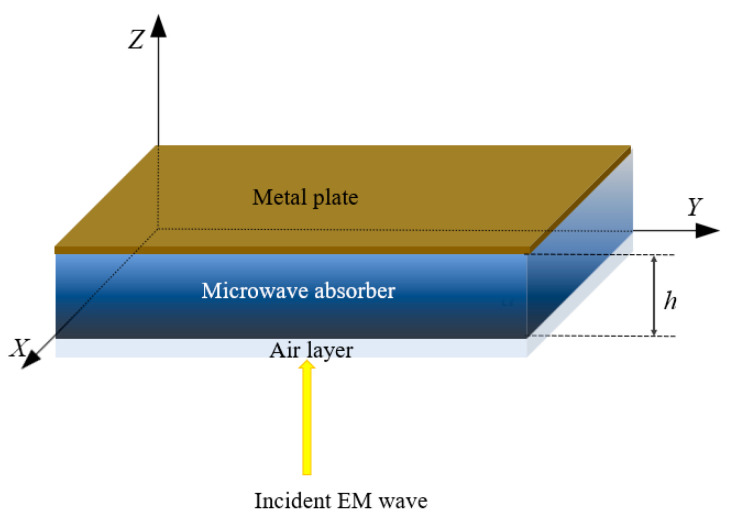
Analytical model of gradient graphene/polymer nanocomposites under microwave irradiation.

To accurately describe the absorbing loss mechanism of composites under electromagnetic radiation, an analysis model was established in this paper based on Maxwell’s electromagnetic theory and effective medium theory. As shown in Figure 1, the composite material is represented as an infinite plate in the *xy* plane with a thickness of *h*. In this analysis, since the graphene/polymer nanocomposite is a lossy medium, the internal electric field (*E*) and magnetic induction intensity (*B*) of the composites must satisfy Maxwell’s equations [27]:

(3) ∇⋅E=0,

(4) ∇⋅B=0,

(5) $$\nabla \times E=-\frac{\partial B}{\partial t},$$

(6) $$\nabla \times B=\mu \varepsilon \frac{\partial E}{\partial t}+\mu \sigma E,$$

in which μ, ε, and σ denote the permeability, dielectric permittivity, and conductivity of the material, respectively.

According to Equations (5) and (6), the electric field (*E*) and magnetic induction intensity (*B*) of gradient composites satisfy the following equations:

(7) $$\nabla^{2}E=\mu \varepsilon \frac{\partial^{2}E}{\partial t^{2}}+\mu \sigma \frac{\partial E}{\partial t},$$

(8) $$\nabla^{2}B=\mu \varepsilon \frac{\partial^{2}B}{\partial t^{2}}+\mu \sigma \frac{\partial B}{\partial t},$$

At the interface between two different media, the material is discontinuous. According to Maxwell’s electromagnetic theory, the electric field and magnetic field at the interface for lossy media satisfy the following boundary conditions:

(9) $$\varepsilon_{1}E_{1}^{⊥}-\varepsilon_{2}E_{2}^{⊥}=0,$$

(10) $$B_{1}^{⊥}-B_{2}^{⊥}=0,$$

(11) $$E_{1}^{∥}-E_{2}^{∥}=0,$$

(12) $$B_{1}^{∥}/\mu_{1}-B_{2}^{∥}/\mu_{2}=0,$$

where $\mu_{1}$ and $\varepsilon_{1}$ are the permeability and dielectric permittivity of material 1, respectively, and $\mu_{2}$ and $\varepsilon_{2}$ are the permeability and dielectric permittivity of material 2, respectively.

The electric field and magnetic induction intensity in the material under microwave radiation can be obtained using Equations (7)–(12). The expression is as follows:

(13) $$E_{i}(z,t)=E_{0i}e^{j({\tilde{k}}_{i}z-\omega t)}=E_{0i}e^{-k_{i2}z}e^{j(k_{i1}z-\omega t)},$$

(14) $$B_{i}(z,t)=\frac{{\tilde{k}}_{i}}{\omega }E,$$

where ω is the angular frequency, the wave number (${\tilde{k}}_{i}$) consists of the real and imaginary parts:

(15) $${\tilde{k}}_{i}=k_{i1}+jk_{i2},$$

(16) $$k_{i1}=\omega \sqrt{\frac{\mu_{i}\varepsilon_{i}}{2}}{\left[\sqrt{1+{\left(\frac{\sigma_{i}}{\varepsilon_{i}\omega }\right)}^{2}}+1\right]}^{1/2},$$

(17) $$k_{i2}=\omega \sqrt{\frac{\mu_{i}\varepsilon_{i}}{2}}{\left[\sqrt{1+{\left(\frac{\sigma_{i}}{\varepsilon_{i}\omega }\right)}^{2}}-1\right]}^{1/2},$$

The subscript *i* can be B, R, T, or A, representing the incident wave, reflected wave, transmitted wave, and absorbed wave, respectively. For *i* = A, ${\tilde{k}}_{i}$, $\mu_{i}$, $\varepsilon_{i}$, $\sigma_{i}$ denote the wave number, permeability, dielectric constant, and conductivity of the graphene/polymer nanocomposite, respectively. When *i* = B, R, T, ${\tilde{k}}_{i}$, $\mu_{i}$, $\varepsilon_{i}$, $\sigma_{i}$ represent the wave number, permeability, dielectric constant, and conductivity of air, respectively. Thus, $\tilde{k}=k_{1}=\omega \sqrt{\mu \varepsilon }$, $k_{2}=0$.

Hence, due to $e^{-k_{i2}z}$, the amplitude of the electromagnetic wave decreases continuously in the propagation of gradient graphene/polymer nanocomposites. In other words, the propagation of electromagnetic waves in materials involves the continuous absorption and attenuation of electromagnetic energy. $k_{i2}$, the imaginary part of ${\tilde{k}}_{i}$, is the prime reason for electromagnetic absorption and is called the attenuation constant.

Since $\frac{\sigma }{\varepsilon \omega }\ll 1$ at the frequency range of 3–18 GHz for graphene/polymer nanocomposites, it can be deduced that $\sqrt{1+{\left(\frac{\sigma }{\varepsilon \omega }\right)}^{2}}\approx 1+\frac{1}{2}{\left(\frac{\sigma }{\varepsilon \omega }\right)}^{2}$. Hence, the attenuation constant can be approximated as

(18) $$k_{2}=\omega \sqrt{\frac{\mu \varepsilon }{2}}{\left[\sqrt{1+{\left(\frac{\sigma }{\varepsilon \omega }\right)}^{2}}-1\right]}^{1/2}\approx \frac{\sigma }{2}\sqrt{\frac{\mu }{\varepsilon }}=\frac{\sqrt{\mu \sigma \omega \tan\delta }}{2},$$

where $\tan\delta =\frac{\varepsilon^{\prime \prime }}{\varepsilon }=\frac{\sigma }{\omega \varepsilon }$ is defined as the dielectric loss angle tangent.

In this case, the electric field in the gradient composites can be approximated as

(19) $$E(z,t)=E_{0}e^{-\frac{\sqrt{\mu \sigma \omega \tan\delta }}{2}z}e^{i(k_{1}z-\omega t)},$$

Based on the Poynting theorem, and combined with the electric field and magnetic induction intensity obtained from Equations (13) and (14), the intensity of the electromagnetic wave can be calculated as

(20) $$I_{i}=(k_{i1}/2\mu \omega )E_{0}^{2}e^{-2k_{i2}z},$$

For absorbing materials, incident wave intensity, reflected wave intensity, transmitted wave intensity, and absorbed wave intensity have the following relationship:

(21) $$I_{R}+I_{T}+I_{A}=I_{B},$$

It can be deduced that the energy of electromagnetic waves absorbed by the material is negatively correlated with the intensity of the reflected wave. Hence, the reflection coefficient (*R*) of the material can be considered an index to reflecting the absorbing performance of the material, which can be calculated as follows:

(22) $$R=\left|\frac{I_{R}}{I_{B}}\right|,$$

Further, *R* can be transformed into reflection loss (RL), a commonly used indicator for evaluating the wave-absorption performance of gradient composites, using the following equation:

(23) RL(dB)=10lgR.

As the main parameter affecting wave absorption performance, the effective dielectric permittivity of gradient graphene/polymer nanocomposites can be obtained based on Maxwell’s effective medium theory, considering the interface effect. It is assumed that the polymer matrix is isotropic and the oblate spheroidal graphene nanosheets are transversely isotropic and randomly oriented. Thus, it can be expressed as [28]

(24) $$\begin{array}{l} (1-\frac{f_{g}(z)}{1-f_{\mathrm{int}}})\frac{\varepsilon_{0}^{*}-\varepsilon_{e}^{*}(z)}{\varepsilon_{e}^{*}(z)+(1/3)(\varepsilon_{0}^{*}-\varepsilon_{e}^{*}(z))}+\\ \frac{f_{g}(z)}{3(1-f_{\mathrm{int}})}[\frac{2(\varepsilon_{1}^{coat*}(z)-\varepsilon_{e}^{*}(z))}{\varepsilon_{e}^{*}(z)+S_{11}(\varepsilon_{1}^{coat*}(z)-\varepsilon_{e}^{*}(z))}+\frac{\varepsilon_{3}^{coat*}(z)-\varepsilon_{e}^{*}(z)}{\varepsilon_{e}^{*}(z)+S_{33}(\varepsilon_{3}^{coat*}(z)-\varepsilon_{e}^{*}(z))}]=0 \end{array},$$

where the complex dielectric permittivity of the polymer matrix is defined as $\varepsilon_{0}^{*}=\varepsilon_{0}+j\sigma_{0}/(\omega \varepsilon_{vac})$ and the complex dielectric permittivity of the gradient composites is defined as $\varepsilon_{e}^{*}(z)=\varepsilon_{e}(z)+j\sigma_{e}(z)/(\omega \varepsilon_{vac})$. $S_{ii}$ (*i* = 1, 2, 3) is the Eshelby tensor related to the shape of the inclusion graphene and can be expressed as follows [29]:

(25) $$\left\{ \begin{array}{l} S_{11}=S_{22}=\frac{\alpha }{2{\left(1-\alpha^{2}\right)}^{3/2}}[\arccos\alpha -\alpha {\left(1-\alpha^{2}\right)}^{1/2}]\\ S_{33}=1-2S_{11} \end{array}\right.,$$

where α is the aspect ratio of graphene and is usually lower than 1.

Since this paper only studies the continuous gradient distribution of graphene concentration along the thickness direction of the plate, it is assumed that the graphene concentration is uniformly distributed in the transverse direction. Based on the fundamental concept of stratification [30], the gradient distribution function of graphene concentration changing continuously along the thickness direction is introduced, as follows:

(26) $$w_{g}(z)={\sum_{i=1}^{n}w_{i}(\frac{z}{h})}^{\lambda_{i}},$$

(27) $$f_{g}(z)=\frac{w_{g}(z)\rho_{m}}{(1-w_{g}(z))\rho_{g}+w_{g}(z)\rho_{m}},$$

where $f_{g}(z)$ and $w_{g}(z)$ are respectively the volume fraction and the mass concentration of graphene; $\rho_{m}$ and $\rho_{g}$ denote the mass density of the matrix and graphene, respectively; $w_{i}$ is the concentration of graphene; *h* is the thickness of the gradient plate; *z* represents the coordinates in the direction of thickness; and $\lambda_{i}$ is the gradient influence factor that controls the form of graphene distribution along with the thickness direction.

For homogeneous composites, $\lambda_{i}=0$. For gradient composites, $\lambda_{i}$ is varied with the different gradient distribution forms.

To account for the influence of the interface effect, a thin interlayer with low conductivity and a dielectric constant is introduced around the graphene filler, forming a coating inclusion with a complex dielectric constant of $\varepsilon_{i}^{coat*}=\varepsilon_{i}^{coat}+j\frac{\sigma_{i}^{coat}}{\omega \varepsilon_{vac}}$. Assuming that the interlayer properties are isotropic, the effective conductivity ($\sigma_{i}^{coat}$) and dielectric permittivity ($\varepsilon_{i}^{coat}$) of the inclusion are expressed as follows [31]:

(28) $$\sigma_{i}^{coat}(z)=\sigma_{fre}^{int}(z)\left[1+\frac{\left(1-f_{int})(\sigma_{i}-\sigma_{fre}^{int}(z)\right)}{f_{int}S_{ii}(\sigma_{i}-\sigma_{fre}^{int}(z))+\sigma_{fre}^{int}(z)}\right],$$

(29) $$\varepsilon_{i}^{coat}(z)=\varepsilon_{fre}^{int}(z)\left[1+\frac{\left(1-f_{int})(\varepsilon_{i}-\varepsilon_{fre}^{int}(z)\right)}{f_{int}S_{ii}(\varepsilon_{i}-\varepsilon_{fre}^{int}(z))+\varepsilon_{fre}^{int}(z)}\right],$$

where the in-plane and out-of-plane complex dielectric permittivity of graphene are defined as $\varepsilon_{1}^{*}=\varepsilon_{1}+j\sigma_{1}/(\omega \varepsilon_{vac})$ and $\varepsilon_{3}^{*}=\varepsilon_{3}+j\sigma_{3}/(\omega \varepsilon_{vac})$, respectively. The volume fraction ($f_{int}$) of the interfacial phase in the inclusion is determined by the thickness ($h_{g}$) of the graphene and the thickness ($h_{int}$) of the interfacial phase, as follows:

(30) $$f_{\mathrm{int}}=1-\frac{h_{int}}{2}(\frac{h_{int}}{2\rho })/(\frac{h_{int}}{2}+h_{g})(\frac{h_{int}}{2\rho }+h_{g}),$$

Because the frequency-dependent electron hopping and dielectric relaxation will affect the dielectric properties of the interface, the influence of the loading frequency is considered, and the conductivity ($\sigma_{fre}^{int}$) and dielectric permittivity ($\varepsilon_{fre}^{int}$) of the interfacial phase are respectively represented as [32,33]

(31) $$\sigma_{\mathrm{int}}^{fre}(z)=\sigma_{\mathrm{int}}^{\prime }(z)\frac{\omega t_{\sigma }{\left(\arctan(\omega t_{\sigma })\right)}^{2}}{{\left[0.5Ln(1+{\left(\omega t_{\sigma }\right)}^{2})\right]}^{2}+{\left(\arctan(\omega t_{\sigma })\right)}^{2}},$$

(32) $$\varepsilon_{\mathrm{int}}^{fre}(z)=\varepsilon_{\mathrm{int}}^{\inf}(z)+\frac{\varepsilon_{\mathrm{int}}^{\prime }(z)-\varepsilon_{\mathrm{int}}^{\inf}(z)}{1+\omega^{2}t_{\varepsilon }^{2}},$$

where $t_{\sigma }$ is the characteristic time of electron tunneling, $t_{\varepsilon }$ is the relaxation time, and $\varepsilon_{\mathrm{int}}^{\inf}$ is the dielectric permittivity of the interface phase at infinite frequency and can be calculated as

(33) $$\varepsilon_{\mathrm{int}}^{\inf}(z)=\varepsilon_{\mathrm{int}}/\tau (f_{g}(z),f^{*},\eta_{\varepsilon }^{\inf}),$$

Due to the interfacial tunneling and Maxwell–Wagner–Sillars (MWS) polarization effect, the electrical conductivity ($\sigma_{int}^{\prime }$) and dielectric permittivity ($\varepsilon_{int}^{\prime }$) of the interface following correction under the static state effect can be calculated as [34,35]

(34) $$\sigma_{int}^{\prime }(z)=\sigma_{int}/\tau (f_{g}(z),f^{*},\eta_{\sigma }),$$

(35) $$\varepsilon_{int}^{\prime }(z)=\varepsilon_{int}/\tau (f_{g}(z),f^{*},\eta_{\varepsilon }),$$

(36) $$\tau (f_{g}(z),f^{*},\eta )=\frac{F(1,f^{*},\eta )-F(f_{g}(z),f^{*},\eta )}{F(1,f^{*},\eta )-F(0,f^{*},\eta )},$$

At the permeability threshold ($f^{*}$), the interface effects increase distinctly with the increase of graphene concentration. To simulate this situation, the Cauchy cumulative probability function (*F*) was introduced:

(37) $$F\left(f_{g}(z),f^{*},\eta \right)=\frac{1}{\pi }\arctan\left(\frac{f_{g}(z)-f^{*}}{\eta }\right)+\frac{1}{2},$$

where η is the scale parameter that characterizes the rate of change when the function (*F*) is near $f=f^{*}$, $\eta_{\varepsilon }^{\inf}$ is the scale parameter for the formation of nanocapacitors at the infinite frequencies, $\eta_{\sigma }$ and $\eta_{\varepsilon }$ represent the scale parameter of the electronic tunneling and the formation of nanocapacitors at the static effect, respectively.

The penetration threshold ($f^{*}$) is related to the shape and size of the graphene filler, denoted as [36]

(38) $$f^{*}=\frac{9S_{33}(1-S_{33})}{-9(S_{33}^{2})+15S_{33}+2}.$$

## 3. Results and Discussion

**Figure 2:**
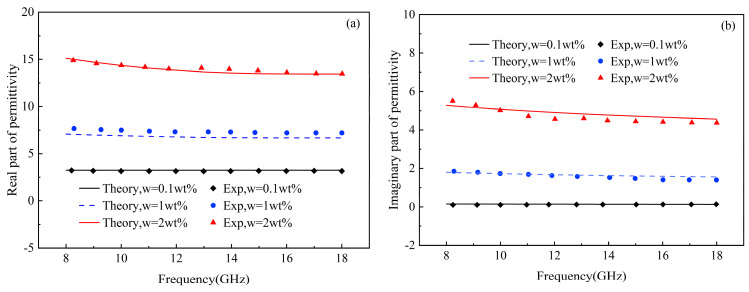
Variation of the equivalent complex permittivity with frequency at different graphene concentrations [15]: (**a**) real part and (**b**) imaginary part of effective dielectric permittivity.

**Figure 3:**
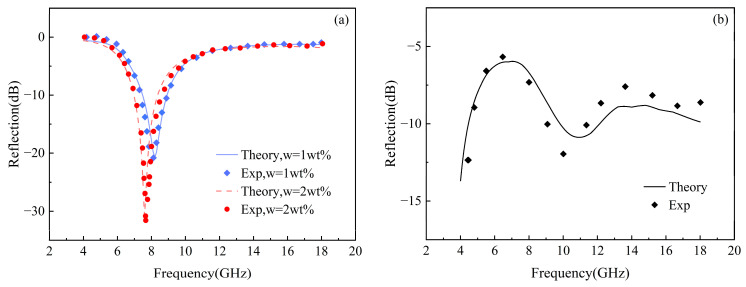
Variation of the reflection loss of the composite material with frequency: (**a**) single-layer absorber [37] and (**b**) triple-layer absorber [38].

To verify the accuracy of the theoretical model established for describing the absorbing properties of graphene/polymer nanocomposites under the influence of the interface effect, the experimental dates were analyzed. The first dataset is the complex dielectric permittivity with varied graphene concentrations reported by De Bellis G et al. [15], and the second dataset includes reflection loss under microwave irradiation for single-layer and triple-layer absorbers studied Li et al. [37] and Zhou et al. [38], respectively. The calculated results and the corresponding experimental data are plotted in Figure 2 and Figure 3. The results indicate that the numerical predictions are in complete agreement with the experimental findings. The physical parameters are as follows: the dielectric permittivity in a vacuum environment is $\varepsilon_{\mathrm{vac}}=8.85\times 10^{-12}\;F/m$, the in-plane and out-of-plane electrical conductivity of graphene are $\sigma_{1}=\sigma_{2}=8.32\times 10^{4}\;S/m$ and $\sigma_{3}=83.2\;S/m$, respectively, the in-plane and out-of-plane dielectric permittivity of graphene are $\varepsilon_{1}=\varepsilon_{2}=30\varepsilon_{vac}$ and $\varepsilon_{3}=0.67\varepsilon_{1}$, respectively. The electrical conductivity and dielectric permittivity of the polymer are $\sigma_{m}=5.5606^{-4}\;S/m$ and $\varepsilon_{m}=3.2\varepsilon_{\mathrm{vac}}$, respectively. The relative permeability of the composites is set to one, due to the negligible magnetic properties of the polymer substrate and graphene. Other parameters are set as shown in Table 1 [15].

**Figure 4:**
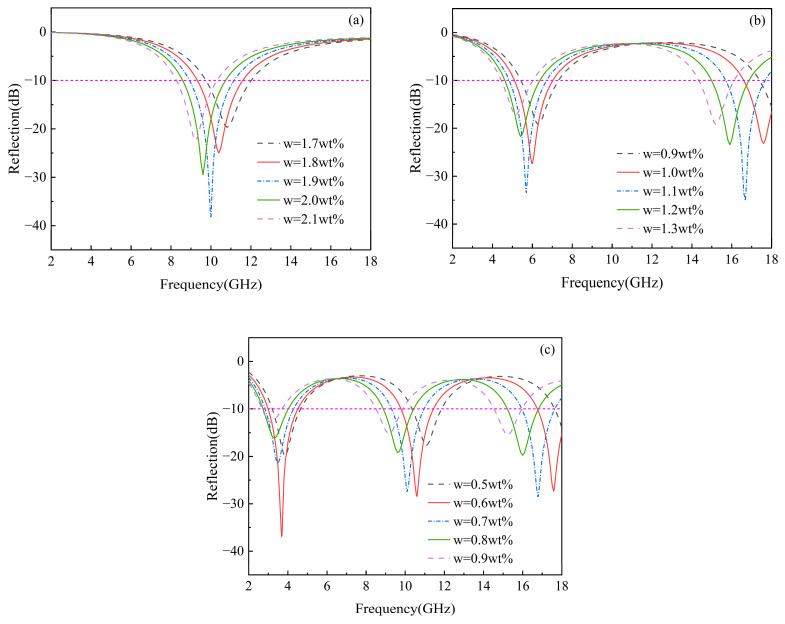
The reflection loss of homogeneous graphene/polymer nanocomposites with different graphene filling concentrations under different plate thicknesses: (**a**) 2 mm, (**b**) 5 mm, and (**c**) 10 mm.

**Figure 5:**
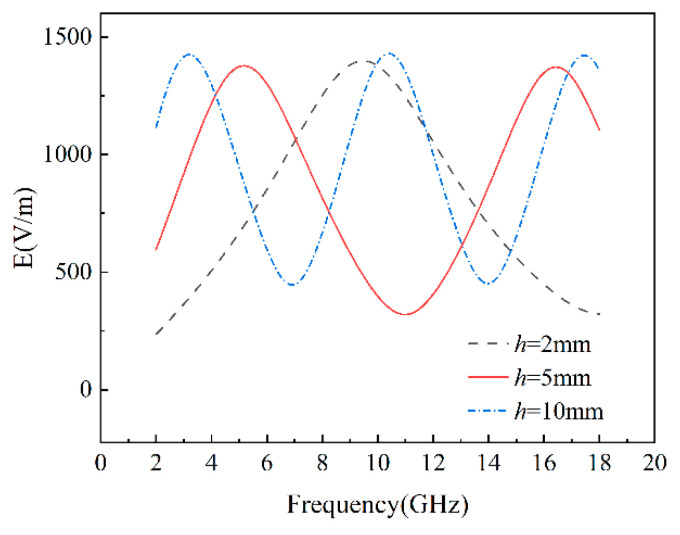
Effect of composites thickness on electric field of homogeneous graphene/polymer nanocomposites with optimal concentration.

**Figure 6:**
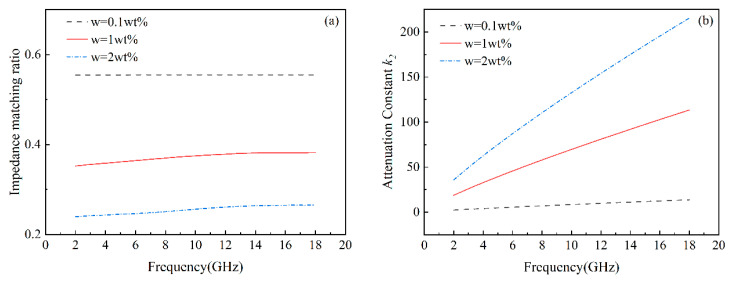
Effect of graphene concentration on (**a**) impedance matching ratio and (**b**) attenuation constant of the composites.

According to Equations (17)–(23), the reflection loss of homogeneous composites is influenced by both the concentration of graphene and the thickness of the composites. Therefore, we simulated the reflection loss for various graphene concentrations and different thicknesses in the 2–18 GHz frequency range, as depicted in Figure 4. The electromagnetic wave was set to vertically incident on the composites. Table 2 lists the minimum reflection loss, effective absorption bandwidth (the frequency range where reflection loss is below −10 dB), and the covered frequency bands. The wave absorption properties depend on the minimum reflection loss and effective absorption bandwidth. For composites with a thickness of 2 mm, the effective bandwidth (RL < −10 dB) is 2.2 GHz, except for a 2.1 wt% graphene concentration, which has a bandwidth of 2.1 GHz, covering the X-band. The 1.9 wt% graphene concentration yielded a minimum reflection loss of −38.14 dB and a peak frequency of 10 GHz. For a thickness of 5 mm, the minimum reflection loss was −35.84 dB at a peak frequency of 16.7 GHz, achieved with a 1.1 wt% graphene concentration. The bandwidth where RL < −10 dB is 3.9 GHz, mainly covering the C and Ku bands. For composites with a thickness of 10 mm, a minimum reflection loss of −36.75 dB was achieved at a graphene concentration of 0.6 wt%, and the effective bandwidth was 4.5 GHz. The maximum effective bandwidth, 4.7 GHz, partially covering the S, C, X, and Ku bands, was achieved at a graphene concentration of 0.7 wt%, with a reflection loss of −28.44 dB. The wave-absorption performance of materials primarily depends on the effective bandwidth and then on the minimum reflection loss. Therefore, for homogeneous composites, the optimal wave-absorption performance was achieved at a graphene concentration of 0.7 wt%. Consequently, it is evident that different thicknesses of graphene/polymer nanocomposites require specific optimal absorbing concentrations to achieve the best performance in the 2–18 GHz frequency range. The optimal concentrations are 1.9, 1.1, and 0.7 wt% for thicknesses of 2, 5, and 10 mm, respectively. As the thickness increases, the wave-absorption performance of homogeneous composites with optimized concentrations improves, and the number of reflection loss peaks increases, aligning with the frequency-dependent electric field curve for composites with optimal graphene concentrations at various thicknesses (Figure 5). On the incident plane, as shown in Figure 5, the frequency and number of electric field peaks are consistent with the reflection loss, since reflection loss is determined by the electric field, according to Equations (13)–(23). It is apparent that the optimal graphene concentration decreases as the composite thickness increases. This occurs because the interface effect causes an increase in graphene concentration in the polymer matrix, reducing the contact distance between graphene nanosheets, forming a capacitor structure, and generating an interfacial tunneling effect, thus increasing the composite’s complex dielectric permittivity (Figure 2). According to Equations (18) and (19), the amplitude of the electromagnetic field in materials is related to the attenuation constant and the material thickness, with the attenuation constant positively correlated with the imaginary part of the dielectric permittivity. Consequently, the attenuation constant increases with higher graphene concentration (Figure 6b). The electric field peak of composites remains around 1400 V/m, regardless of the composite thickness (Figure 5). Thus, the incident electromagnetic field energy is scarcely affected. Therefore, when the graphene concentration is not high enough, it is necessary to increase the composite thickness to achieve strong absorption of the incident electric and magnetic fields. When the graphene concentration is sufficient, electromagnetic wave attenuation can be achieved with a low thickness. Furthermore, as the graphene concentration increases, the reflection loss peak of the composites initially decreases, then increases and shifts from high to low frequency (Figure 4). This is attributed to the high conductivity of graphene. Near the optimal concentration range, increasing the graphene concentration reduces the impedance matching performance, while decreasing the graphene concentration weakens the dielectric loss capability (Figure 6). Equations (18) and (19) show that for homogeneous graphene/polymer nanocomposites with the same thickness, the peak position is inversely proportional to the dielectric properties of the composites. As the graphene concentration increases, the complex dielectric permittivity of the composite material rises, shifting the peak from high to low frequency.

**Figure 7:**
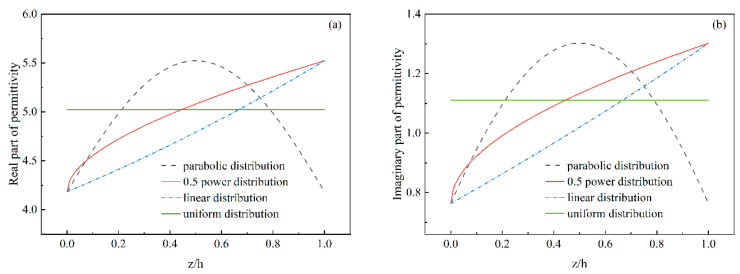
Variation of the equivalent complex dielectric permittivity along the thickness direction of the composites with a loading frequency of 10 GHz for different distribution models of graphene concentration: (**a**) real part and (**b**) imaginary part of effective dielectric permittivity.

Based on the results shown in Figure 6, the surface concentration of graphene in the matching layer should be lower in the design of gradient distribution, while the concentration in the middle and bottom layers can be adjusted to enhance impedance matching and dielectric loss capacity. Thus, three gradient distribution models—linear, parabolic, and 0.5 power distribution—were adopted, with the electromagnetic wave incident on the surface with low graphene concentration, as shown in Figure 7. The distribution of complex dielectric permittivity aligns with the graphene concentration model in the thickness direction for gradient composites within a concentration range of 0.5–0.8 wt%. Analysis of homogeneous composites shows that optimal graphene concentrations vary with thickness for achieving the best wave absorption. Therefore, the gradient absorber structure was designed based on this conclusion. For a material thickness of 2 mm, the concentration ranges are 1.7–2.0 wt%, 1.8–2.0 wt%, and 1.9–2.0 wt% for parabolic, 0.5 power, and linear distributions, respectively. For a thickness of 5 mm, the ranges are 0.9–1.2 wt% for both parabolic and 0.5 power distributions, and 1.0–1.2 wt% for the linear distribution. When the thickness is 10 mm, the concentration range for all three gradient models is 0.5–0.8 wt%.

**Figure 8:**
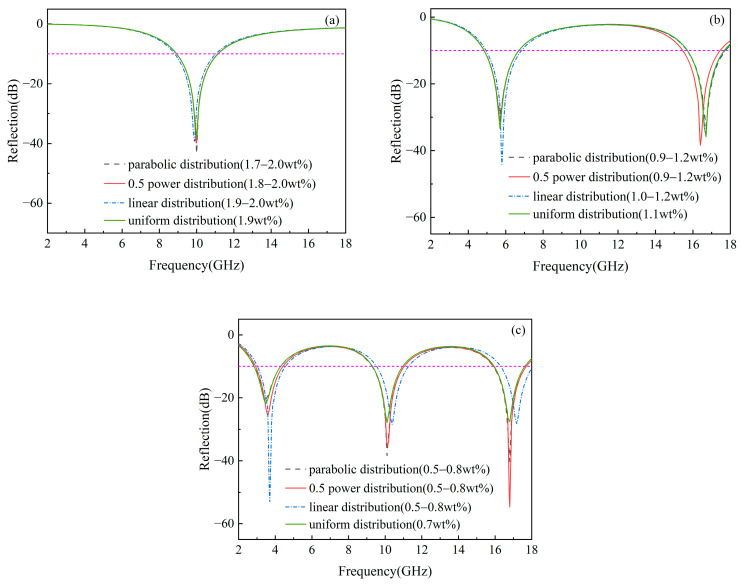
Effect of gradient distribution type on reflection loss of gradient composites with different thicknesses: (**a**) 2 mm, (**b**) 5 mm, and (**c**) 10 mm.

**Figure 9:**
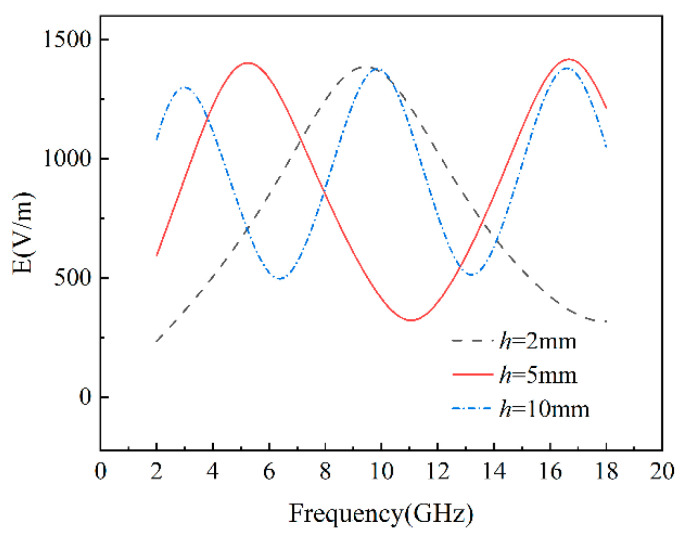
Effect of composites thickness on electric field of gradient graphene/polymer nanocomposites with parabolic distribution.

The wave-absorption properties of gradient composites with varying thicknesses and graphene concentration ranges across different distribution patterns in the 2–18 GHz frequency range are further investigated, as shown in Figure 8. Table 3 lists the minimum reflection loss and effective bandwidth for the gradient-distributed composites. With increasing thickness, the number of reflection loss peaks increases due to the corresponding increase in the number of electric field peaks, as illustrated in Figure 9, since reflection loss is influenced by the electric field. Additionally, the results in Figure 8 indicate that all graphene concentration distribution patterns enhance the wave-absorption performance of gradient composites. Particularly, with a material thickness of 10 mm, the 0.5 power distribution of graphene concentration yields the broadest effective bandwidth of 5.2 GHz and the lowest RL of −54.7 dB at 16.8 GHz in the Ku band. For linear and parabolic distributions, the effective bandwidths are 5 GHz and 5.1 GHz, respectively, with minimum reflection losses of −52.99 dB at 3.7 GHz in the S band and −40.21 dB at 16.8 GHz in the Ku band. However, homogeneous composites exhibit an effective bandwidth of only 4.7 GHz and a minimum RL of −28.44 dB. Notably, when the thickness of the gradient composites is 10 mm, different graphene distribution models enhance absorption performance in specific bands, namely Ku for parabolic, Ku for 0.5 power, and S for linear distribution (Figure 8). Consequently, it can be concluded that gradient-distributed graphene concentration improves the wave-absorption performance of graphene/polymer nanocomposites. This improvement occurs because a gradient distribution of graphene concentration allows the surface with lower graphene concentration to achieve better impedance matching, and higher concentrations are achieved more rapidly in the thickness direction compared to uniform distribution, as depicted in Figure 7. The increased graphene concentration enhances the electrical conductivity and dielectric permittivity of the composites, leading to higher energy dissipation (Figure 2 and Figure 6b), causing electromagnetic waves to be quickly absorbed in the middle and lower sections, thus enhancing the material’s wave-absorption performance. When using parabolic, 0.5 power, and linear distributions, the concentration on the incident surface increases sequentially, and as the material thickness grows, the concentration tends to equalize (Figure 8). This phenomenon occurs because, for a consistent graphene concentration range, the composite dielectric permittivity of gradient composites decreases sequentially from the middle to the bottom of the material with this distribution order, as shown in Figure 7. To achieve a high concentration more rapidly and increase electromagnetic wave loss, the incident surface concentration must increase sequentially. As the electromagnetic field propagates through the material, it decays gradually, and the electromagnetic field is related to the complex dielectric permittivity and thickness of the gradient composites (Equations (18) and (19)). The dielectric permittivity is inversely related to the material thickness, and the electric field peaks are consistent across materials of different thicknesses (Figure 9). Therefore, when the material is sufficiently thick, the incident surface concentration need not increase sequentially to achieve effective absorption.

**Figure 10:**
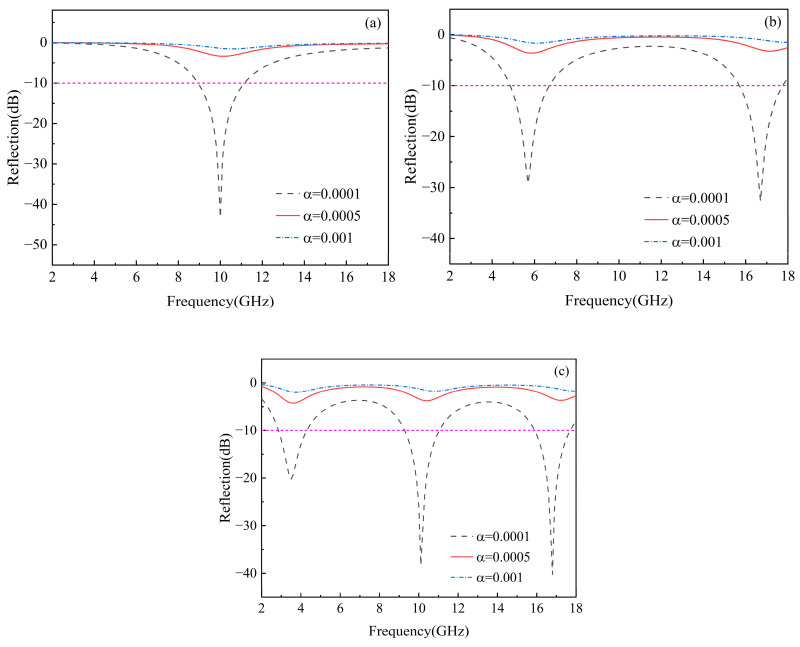
Effect of aspect ratio of graphene on reflection loss of gradient-distributed composites with different thickness under parabolic distribution: (**a**) 2 mm, (**b**) 5 mm, and (**c**) 10 mm.

The aspect ratio of graphene is a crucial factor influencing its dielectric properties and can affect the wave-absorption performance of gradient-distributed composites (Equations (24) and (25)). Therefore, the effect of the aspect ratio of graphene on the reflection loss of gradient composites with a parabolic distribution at different thicknesses was studied, as shown in Figure 10. As the aspect ratio of graphene increases, the minimum reflection loss of the gradient composites gradually increases, and the effective bandwidth decreases. The distance between graphene nanosheets shortens with the increase in the aspect ratio, resulting in a reduced electron tunneling effect at the interface and reducing the possibility of forming nanocapacitors at the interface. Consequently, the conductive graphene network is scarcely produced, leading to a decrease in the effective dielectric permittivity and conductivity of the gradient composites, resulting in poorer dielectric loss performance. Therefore, when the aspect ratio of graphene changes from 0.0001 to 0.001, the dielectric properties of graphene significantly deteriorate, resulting in very poor microwave absorption performance. Additionally, with the increase in the aspect ratio of graphene, the reflection loss peak of the composite gradually shifts from low to high frequency, and the number of reflection loss peaks decreases.

## 4. Conclusions

In this paper, an analytical model of wave absorption analysis was constructed based on Maxwell’s theory of electromagnetism combined with the equivalent performance characterization theory of gradient graphene/polymer nanocomposites to explore absorbing properties. The comparison between this model and the experiment verified its applicability to microwave reflection problems. Three gradient structures under three plate thicknesses were designed based on the optimal concentration of the combined homogeneous composites under different plate thicknesses. The absorbing properties of the gradient composites increased with the increase in thickness, and the gradient distribution was parabolic distribution, 0.5 power distribution, and linear distribution; the concentration of the incident surface increased in turn, and as the thickness of the material increased, the concentration tended to be equal. In addition, the absorbing properties of the gradient composites decreased with the increase of the aspect ratio of graphene. The structural design of continuous gradient distribution not only led to the improvement of impedance matching of the composite materials but also enhanced the attenuation ability of electromagnetic waves, resulting in the better absorbing performance of gradient composites than that of homogeneous composites. As shown above, the analytical model was used to explore the influence of the gradient structure of graphene/polymer nanocomposites on the microwave absorption performance, which makes a certain theoretical contribution to the development of this high-performance and lightweight stealth material in aerospace and military applications and in other fields.

## Figures and Tables

**Table 1:** Set value for parameters of graphene/polymer nanocomposites during numerical calculation [15].

Parameter	Value
Aspect ratio of graphene α	1 × 10^−4^
Thickness of graphene $h_{g}$(m)	3 × 10^−9^
Thickness of the interlayer $h_{int}$(m)	0.9 × 10^−9^
Scale parameters of the formation nanocapacitor at the static state $\eta_{\varepsilon }$	2.8 × 10^−13^
Scale parameter of the electron tunneling at the static state $\eta_{\sigma }$	9 × 10^−3^
Scale parameters of the formation nanocapacitors at the infinite frequencies $\eta_{\varepsilon }^{\inf}$	3.7 × 10^−5^
Characteristic time of electron tunneling $t_{\sigma }$(s)	1 × 10^−6^
Relaxation time $t_{\varepsilon }$(s)	2 × 10^−3^

**Table 2:** Wave-absorption performance of homogeneous graphene/polymer nanocomposites.

Thickness	Performance	Concentration of Graphene (wt%)				
		**1.7**	**1.8**	**1.9**	**2.0**	**2.1**
2 mm	Peak value of RL (dB)	−19.66	−24.91	−38.14	−29.5	−22.18
	Frequency of RL peak (GHz)	10.8	10.4	10	9.6	9.2
	Effective bandwidth (dB)	2.2	2.2	2.2	2.2	2.1
	Covered band of effective bandwidth (1.9 wt%)	X (8.9–11.1 GHz)				
		**0.9**	**1.0**	**1.1**	**1.2**	**1.3**
5 mm	Peak value of RL (dB)	−19.17	−27.36	−35.84	−23.43	−19.12
	Frequency of RL peak (GHz)	6.3	6	16.7	15.9	15.2
	Effective bandwidth (dB)	2.6	3.2	3.9	3.7	3.4
	Covered band of effective bandwidth (1.1 wt%)	C (4.9–6.8 GHz), Ku (15.7–17.7 GHz)				
		**0.5**	**0.6**	**0.7**	**0.8**	**0.9**
10 mm	Peak value of RL (dB)	−19.82	−36.75	−28.44	−19.76	−15.43
	Frequency of RL peak (GHz)	3.9	3.7	16.8	16	15.2
	Effective bandwidth (dB)	3.4	4.5	4.7	4.6	3.7
	Covered band of effective bandwidth (0.7 wt%)	S (2.9–4.0 GHz), C (4–4.3 GHz), X (9.3–10.9 GHz), Ku (16–17.7 GHz)				

**Table 3:** The wave-absorption performance of graphene/polymer nanocomposites with gradient-distributed graphene.

Thickness	Performance	Gradient Distribution Type			
		Parabolic Distribution	0.5 Power Distribution	Linear Distribution	Uniform Distribution
2 mm	Peak value of RL (dB)	−43.27	−39.81	−38.9	−38.14
	Frequency of RL peak (GHz)	10	10	9.9	10
	Effective bandwidth (dB)	2.2	2.2	2.2	2.2
	Covered band of effective bandwidth (parabolic distribution)	X (8.9–11.1 GHz)			
5 mm	Peak value of RL (dB)	−32.7	−38.37	−44.18	−35.84
	Frequency of RL peak (GHz)	16.7	16.4	5.8	16.7
	Effective bandwidth (dB)	4	3.9	4	3.9
	Covered band of effective bandwidth (parabolic distribution)	C (4.9–6.8 GHz), Ku (16–18 GHz)			
10 mm	Peak value of RL (dB)	−40.21	−54.7	−52.99	−28.44
	Frequency of RL peak (GHz)	16.8	16.8	3.7	16.8
	Effective bandwidth (dB)	5.1	5.2	5	4.7
	Covered band of effective bandwidth (parabolic distribution)	S (2.9–4.0 GHz), C (4–4.3 GHz), X (9.3–11.1 GHz), Ku (15.8–17.7 GHz)			

## Data Availability

Data are contained within the article.
